# “Time to See” in Infantile Nystagmus

**DOI:** 10.1167/iovs.67.5.17

**Published:** 2026-05-07

**Authors:** Katherine Ward, Lee McIlreavy, Matt J. Dunn, Christopher M. Harris, Fergal A. Ennis, Jonathan T. Erichsen

**Affiliations:** 1 School of Optometry and Vision Sciences, Cardiff University, Cardiff, United Kingdom; 2 Royal Eye Infirmary, Derriford Hospital, Plymouth, United Kingdom

**Keywords:** infantile nystagmus, viewing time, psychophysics

## Abstract

**Purpose:**

Interventions that dampen the involuntary eye oscillation in infantile nystagmus (IN) often only elicit modest improvement in visual acuity (VA), even when subjective improvement is reported. Unique to nystagmus, the repeated movement of the fovea away from the intended visual target introduces a temporal dimension to vision. We hypothesized that the viewing time required to resolve optotype stimuli is increased in people with IN and that this measure of “time to see” increases with IN severity.

**Methods:**

Exposure duration thresholds (viewing times) were measured psychophysically for 18 individuals with IN and 14 controls for an optotype orientation discrimination task at spatial frequencies varying relative to each participant's VA. Viewing times were measured at two gaze positions (toward the IN null zone, where waveform intensity is lower, and away from the null zone, where intensity is higher) to establish the effect of IN severity as quantified by intensity.

**Results:**

Exposure duration thresholds were significantly longer in those with IN than controls (*P* = 0.027) and increased significantly with IN intensity (*P* = 0.034). Both effects were greatest for optotypes sized at participant-relative VA.

**Conclusions:**

“Time to See” is increased in IN and is sensitive to within-participant changes in IN severity, suggesting that exposure duration threshold could be a useful measure of visual function in people with IN.

The time required for visual processing of a stimulus, or “time to see,” is well understood in the typical visual system. Stimulus detection requires a threshold of luminous energy to be reached within a critical duration over which temporal summation occurs. When stimulus exposure duration is at or below this critical window, this relationship follows Bloch's law,^1^^,^^2^ which states that the luminance required for detection is inversely proportional to stimulus duration.

This temporal relationship also applies to resolution. Visual acuity (VA) is reduced at very short stimulus durations, improves with viewing time, and reaches a plateau beyond a critical duration. In typically sighted adults under photopic conditions, this critical duration is reported to range from approximately 250 to 620 ms for optotype stimuli,^3^^–^^7^ although some studies have found continued VA improvements up to 1000 ms^8^^–^^10^ or even 10,000 ms.^11^ These findings indicate that adequate viewing time is essential for achieving maximum resolution.

The present study investigated “time to see” in people with infantile nystagmus (IN), a condition characterized by continuous oscillatory eye movements, usually in the horizontal axis.^12^ The temporal relationship between VA and viewing time has not yet been established in this condition as it has in typically sighted individuals. During an oscillatory cycle of IN, there is often a short foveation period during which the photoreceptor-dense fovea remains relatively close to the target and eye velocity is relatively low.^13^^,^^14^ The fovea then drifts away from the target with increasing velocity before returning to it. As a result, the duration during which high-resolution foveal viewing is possible is restricted, limiting the useful exposure time for resolving stimuli within each oscillation cycle.

Anecdotally, individuals with IN often describe being “slow to see,” and response times for VA-level stimuli have been shown to be longer in those with IN relative to controls.^15^^,^^16^ Reaction times to novel stimuli are also delayed in IN^17^^,^^18^; however, when fixation is achieved, visual processing speed itself is comparable to that of typically sighted individuals.^18^^,^^19^ This suggests that delays are primarily due to the time taken to locate or foveate, rather than process, stimuli. For example, if stimulus onset occurs just after a foveation period, the stimulus may not be viewed with sufficient detail until the next foveation. For more complex or less salient stimuli, multiple foveations may be required to accumulate sufficient visual information.^3^

IN is associated with reduced VA,^12^^,^^20^ although the precise contribution of oscillatory waveform characteristics to this visual impairment is not fully understood. Between individuals, VA correlates with waveform features; those with lower nystagmus amplitude and longer and more stable foveation periods tend to have better VA.^21^^–^^23^

Waveform features can be modified within individuals—for example, through gaze position. A common feature of IN is a null zone, a region of gaze in which the IN oscillation is dampened, typically having lower amplitude and frequency (and thus intensity, defined as the product of the two), as well as longer and more positionally stable foveation periods.^21^ The null zone is often used preferentially to improve visual performance to the extent that, although the null zone tends to lie within 10° either side of the primary position, those with an eccentric null may adopt an anomalous head posture to place their eyes in this region.^12^ IN interventions are also available that aim to dampen the waveform in order to improve vision.^24^

However, such modification of an individual's waveform may only elicit modest changes in VA, even when subjective visual improvement is reported.^25^^,^^26^ This suggests that conventional VA measures may not fully capture the functional impact of the IN oscillation and so may only partially reflect the visual experience of those with IN, and, crucially, may not be an appropriate outcome measure to fully evaluate the impact of interventions and treatments that aim to dampen nystagmus.^25^

VA is typically measured with unrestricted viewing times and so may be insensitive to temporal limitations imposed by nystagmus oscillations. Incorporating a temporal constraint into visual assessment may therefore provide a more sensitive measure of functional vision in IN. In fact, previous findings of increased response times when IN intensity is increased through stress,^16^ as well as time-restricted VA being worse at eccentric gaze positions (where IN tends to be exacerbated),^27^ suggest that temporal vision is affected by changes in waveform.

This study builds upon the above work by directly quantifying the exposure duration required to resolve stimuli in individuals with and without IN. Measuring exposure duration threshold rather than response time eliminates potential conflation among motor, sensory, and cognitive aspects of the response. Studies have reported reduced exposure duration thresholds in some, but not all, participants following nystagmus-dampening surgeries,^28^^,^^29^ although methodological limitations such as unavoidable order effects complicate interpretation. We therefore aimed to validate these results, overcoming the effects of order through use of reversible gaze-induced waveform changes.

To examine the potential of exposure duration threshold as a method of visual assessment and an outcome measure in IN, as well as to characterize more fully the relationship between exposure duration threshold and spatial frequency in IN, three hypotheses were tested:

1. Exposure duration thresholds are greater in IN than in typical observers, consistent with reports of increased “time to see.”
2. Exposure duration threshold increases under conditions of exacerbated IN (i.e., increased IN intensity, elicited through changing gaze position).
3. Higher spatial frequencies require longer stimulus exposure durations in both IN and typically sighted participants.

## Methods

### Ethics

This study complied with the tenets of the Declaration of Helsinki and was approved by the Research Ethics Committee of the School of Optometry and Vision Sciences, Cardiff University (project no. 1563). Informed written consent was obtained from participants after explanation of the nature and possible consequences of the study.

### Participants

Eighteen individuals with IN (seven females; 20–66 years; mean age = 44 years) and 14 typically sighted individuals (nine females; 19–65 years; mean age = 41 years) participated. Clinical and oculomotor characteristics of participants with IN are presented in Table 1. All participants underwent preliminary clinical testing including slit-lamp examination, indirect ophthalmoscopy, and optical coherence tomography. Clinical oculomotor assessment and alternating cover tests were performed to rule out binocular vision abnormalities in control participants and, for those with IN, to approximately identify the null zone and confirm that nystagmus beat direction did not reverse upon occlusion (ruling out fusion maldevelopment nystagmus syndrome).

**Table 1.** Clinical Characteristics of Participants With IN

					Primary Position Waveform		
Participant	Age (y)/ Sex (M/F)	Diagnosis	Test Eye Refraction (Lens Type)	Test Eye Clinical VA (logMAR)	Type	Amplitude (°)	Frequency (Hz)
N1	38/M	Idiopathic	+3.50/−3.25 × 30 (TL)	0.42	BDJR	1.04	3.64
N5	63/M	Idiopathic	−7.50/−1.00 × 35 (TL)	0.52	JR_EF_	1.71	4.98
N10	57/M	Congenital cataracts	+10.00 DS (CL)	0.42	JL_FS_	1.92	2.72
N12	58/M	OCA type 2	+2.00/−1.00 × 180 (TL)	0.34	JR	1.55	2.35
N13	20/F	Ocular albinism	+0.75/−2.75 × 175 (TL)	0.42	JL_EF_	4.04	3.04
N14	65/M	Idiopathic	−11.25/−3.25 × 90 (TL)	0.20	JL_EF_	5.37	4.85
N15	23/M	Idiopathic	+6.25/−2.25 × 85 (CL)	0.36	JL	7.89	4.02
N16	58/F	Idiopathic	+4.00/−4.75 × 16 (TL)	0.42	DJR	2.55	4.88
N17	44/F	Congenital cataract	+8.00 DS (CL)	0.80	P_FS_	2.63	4.06
N18	46/M	Congenital cataract	+11.25/−2.25 × 110 (TL)	0.50	JL_EF_	1.36	4.02
N19	20/F	Idiopathic	−2.25/−1.25 × 100 (TL)	0.12	JR	11.22	4.23
N20	66/M	Idiopathic	−0.50/−0.25 × 60 (N/A)	0.20	JL_EF_	4.93	4.04
N21	58/M	Idiopathic (*PAX6*)	−5.75/−1.00 × 165 (TL)	0.44	P_FS_	0.57	1.90
N22	53/M	OCA (type unknown)	+8.50/−2.75 × 160 (TL)	0.60	JR_EF_	3.19	3.48
N26	33/F	Idiopathic	+2.00/−5.00 × 151 (TL)	0.30	PP_FS_	5.78	3.02
N28	25/M	Optic nerve hypoplasia	−0.75/−6.00 × 25 (TL)	0.26	JL_EF_	4.98	2.86
N29	23/F	OCA (type unknown)	+2.00/−2.50 × 25 (TL)	0.70	JL_FS_	10.5	2.81
N31	32/F	Idiopathic	+3.00 DS (TL)	0.56	JL_EF_	6.82	4.48

BDJR, bidirectional jerk (right); CL, contact lenses; DJR, dual jerk (right); DS, diopter sphere; F, female; JL_EF_, jerk (left) with extended foveations; JL, pure jerk (left); JL_FS_, jerk (left) with foveating saccades; JR_EF_, jerk (right) with extended foveation; JR_EF_, jerk (right) with extended foveation; JR, pure jerk (right); M, male; N/A, not applicable; OCA, oculocutaneous albinism; P_FS_, pendular (with foveating saccades); PP_FS_, pseudo-pendular (with foveating saccades); *PAX6*, paired box 6; TL, trial lenses.

Refraction was performed by an optometrist, following which clinical VA was measured for all participants at 3 meters using a backlit Early Treatment of Diabetic Retinopathy Study (ETDRS) chart (Precision Vision; Woodstock, IL, USA). Participants used their preferred gaze position, and viewing time was unrestricted. Diagnosis of IN as reported by the participant or their ophthalmologist was confirmed by high-speed eye movement recording to verify the presence of accelerating slow phases. Control participants were only included if their corrected VA was 0.00 logMAR or better. Any participants with ocular abnormalities (other than those associated with IN) or with systemic conditions or medications that may affect vision were excluded.

To prevent visual distortions from off-axis viewing, participants who had up-to-date contact lenses were encouraged to wear these for the experiment. Otherwise, refractive correction was provided using full-aperture trial lenses in an adjustable custom lens holder that enabled alignment of the trial lenses with the pupil across a range of gaze positions. Habitual prescription was used unless a difference of greater than ±0.50 D mean sphere was found during refraction, in which case the new prescription was provided.

The experiment was performed monocularly, with the test eye being the eye with the best clinical VA. In cases of equal VA, the dominant eye was used. Ocular dominance was assessed using the +1.00 DS sensory blur test and, if inconclusive, was confirmed using the distance “hole-in-card” motor test.^30^ The non–test eye was occluded using an eye patch.

### Apparatus

**Figure 1.**
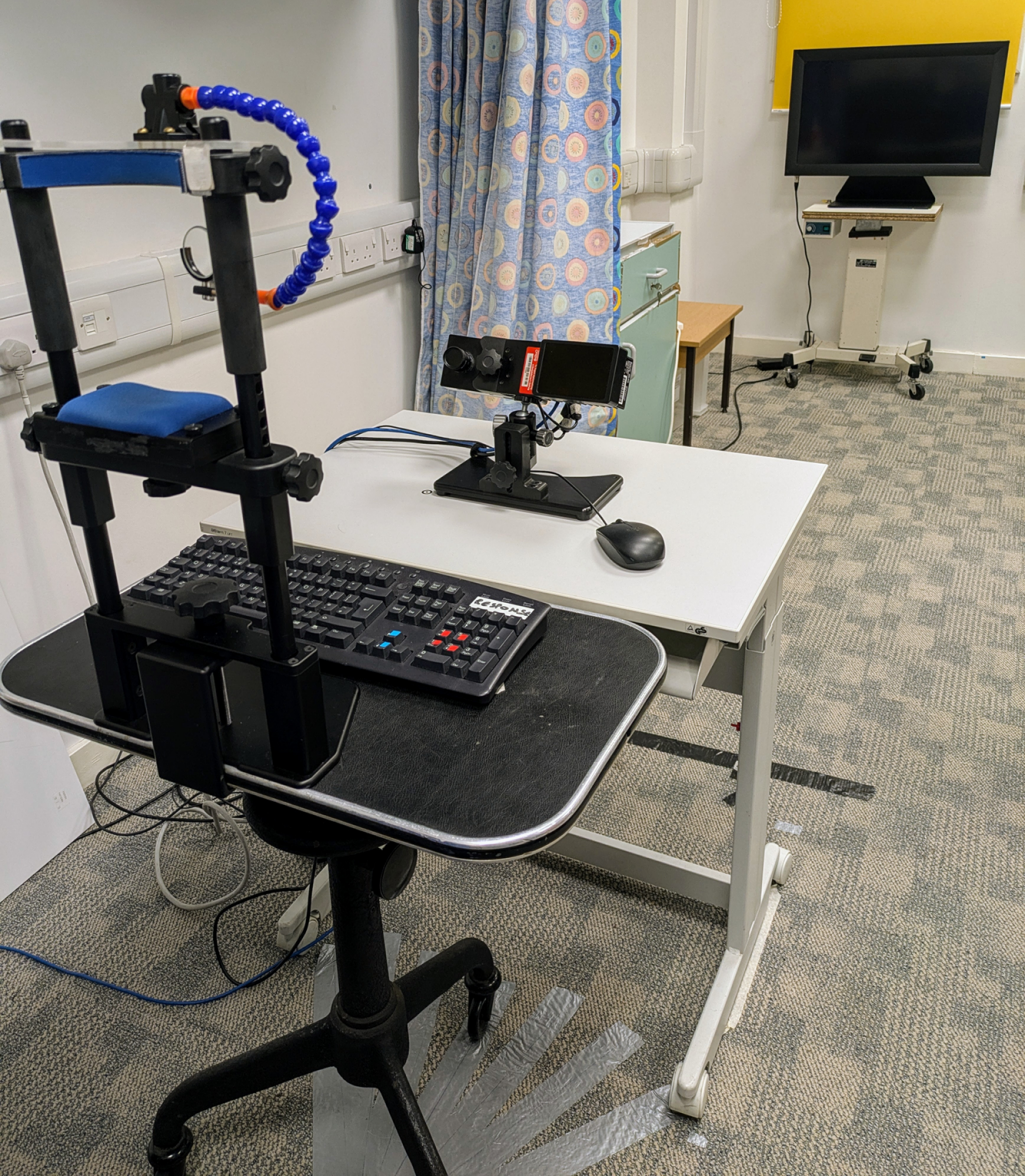
Experimental setup showing eye tracker, display monitor, adjustable lens holder, and floor markings to guide rotation of the chin rest table.

The experimental setup is shown in Figure 1. Stimuli were rendered using a GeForce RTX 2060 graphics card (NVIDIA, Santa Clara, CA, USA) and displayed on a 708 × 400-mm liquid-crystal display (LCD) monitor (Display++; Cambridge Research Systems, Rochester, UK) with a resolution of 1920 × 1080 pixels, refresh rate of 120 Hz, and minimum–maximum luminance range of 0.15 to 102.23 cd/m^2^. Participants sat with their head supported by a chin and forehead rest. The chair and chin rest could be rotated to achieve non–primary gaze positions.

An EyeLink 1000 Plus video-based eye tracker (SR Research, Ottawa, ON, Canada; firmware version 5.12) in desktop configuration was used to record horizontal and vertical monocular gaze position of the test eye at 1000 Hz. The experiment was performed under maximum room lighting, corresponding to 201 lux in the approximate location of participants’ eyes. Psychophysical tests were programmed using the Psychophysics Toolbox extensions^31^^–^^33^ for MATLAB 2018b (MathWorks, Natick, MA, USA). Where aspects of the procedure were carried out in randomized order, this order was determined using the MATLAB randperm function. Responses were recorded using a computer keyboard.

### Procedure

To examine the effect of gaze-related changes in IN waveform on “time to see,” each participant performed the experiment once in primary position and once with the eyes in an eccentric gaze position either 30° to the left or 30° to the right. A broad range of VAs are found in IN, so, to equalize visibility in order for results to be comparable between participants, exposure duration thresholds were measured at spatial frequencies relative to each participant's VA.

The eccentric gaze position used for each participant with IN was determined from eye movement recordings of their test eye at these three locations. First, viewing distance for the display monitor was set to 1 meter, and the EyeLink 1000 Plus was calibrated to the typically sighted researcher using the built-in EyeLink nine-point calibration and validation procedures, with acceptance criteria of worst point error < 1.5° and average error < 1.0°. These procedures require steady fixation of the calibration targets so are often unsuccessful in participants with IN. Where standard calibration is not possible, calibration to a typically sighted observer has been reported to give relative higher accuracy compared to alternative real-time methods.^34^ This approach has also been used in similar studies involving IN cohorts.^35^^,^^36^ Because the eye movement recordings were to be used for assessing relative gaze position as opposed to absolute gaze position, calibration to a typically sighted observer was deemed sufficient.

Following calibration, participants were directed to fixate 1° targets^37^ shown for 10 seconds at the screen center and 30° to the right and left in random order. Participants faced straight ahead throughout and turned their eyes to view the stimuli. Analysis of the recordings to calculate waveform intensity at each position was performed as described by Dunn et al.^38^ To maximize the effect of gaze position, whichever of the two eccentric positions had the greatest difference in intensity from primary position, whether lower or higher, was used. In the event that eye movements could not be recorded with sufficient quality for analysis, as was the case for participants N1, N22, and N29, the eccentric position was determined based on clinical examination of nystagmus intensity.

The two gaze positions tested for each participant are hereafter referred to as *toward-null* and *away-from-null*, with the toward-null position having the lower intensity of the two. Note that the toward-null position may have been either primary position or the eccentric gaze position. For control participants, the eccentric gaze position was chosen so that the proportion of participants tested at each position (right or left) was similar across the two participant groups. To maintain consistent terminology between the cohorts, the central and eccentric gaze positions in the control cohort are referred to as the toward-null and away-from-null positions, respectively.

**Figure 2.**
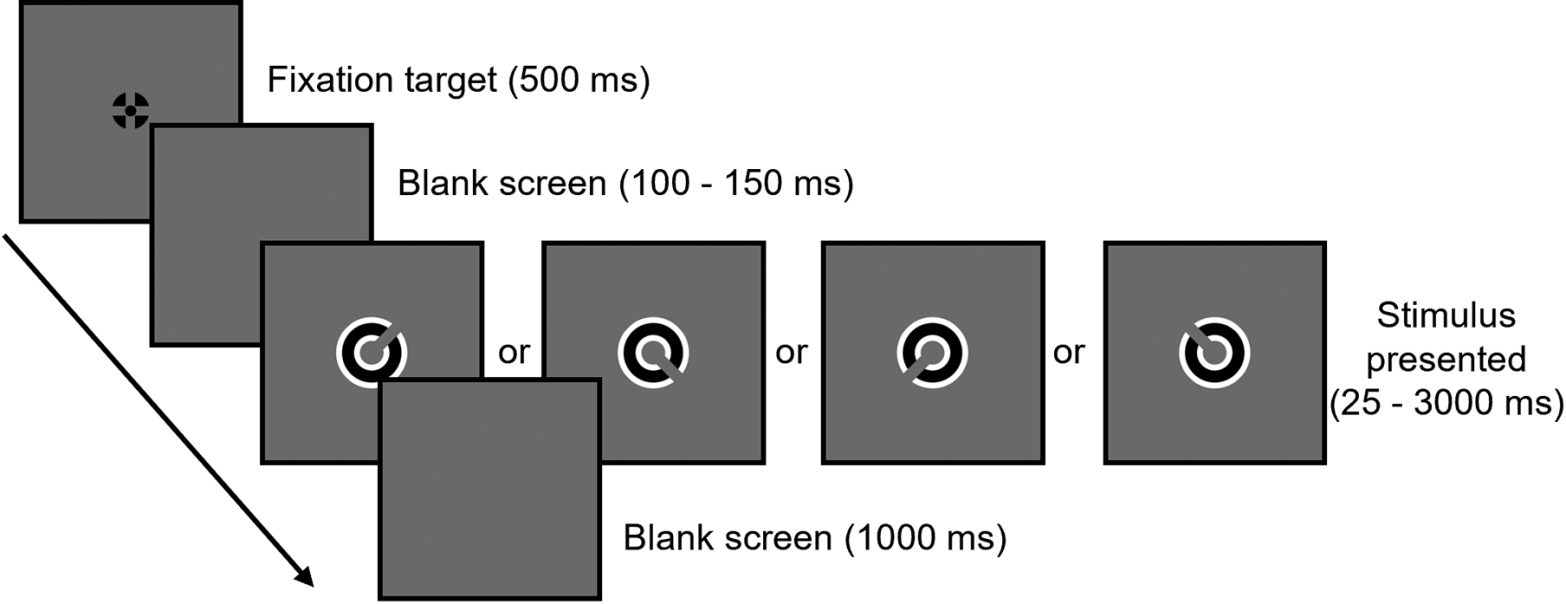
Trial sequence (stimuli not to scale).

VA and exposure duration thresholds were measured using a four-alternative forced-choice paradigm, in which participants identified the gap in a vanishing Landolt C stimulus (shown in Fig. 2) as being in one of the four diagonal orientations (45°, 135°, 225°, or 315°). Oblique orientations were chosen to ensure equal visibility, given the discrepancy between VA in the horizontal and vertical meridians in IN.^39^^,^^40^ A viewing distance of 4 meters was used to achieve sufficient resolution for display of VA-level stimuli. Each trial began with a 0.2° black fixation target^37^ being displayed at the center of the screen against a mid-gray background for 500 ms. The fixation target was extinguished for a random duration between 100 to 150 ms before being replaced with the vanishing Landolt C stimulus. After a set duration, the stimulus was extinguished, and a beep prompted the participant to estimate the stimulus orientation via a button press. There was no time restriction for participants to respond. After the response was made, there was a 1000-ms interval before the next trial began. This sequence is illustrated in Figure 2.

Stimulus sequence was determined using a two-down/one-up psychophysical staircase procedure. The staircase terminated after whichever came first of 12 reversals or 100 presentations. Two staircase step sizes were used: By default, the “small” step size was applied, except in cases where the two most recent steps were in the same direction, in which case the “large” step size was used. The chosen step sizes and stimulus ranges for both VA and exposure duration thresholds were determined by pilot testing.

**Figure 3.**
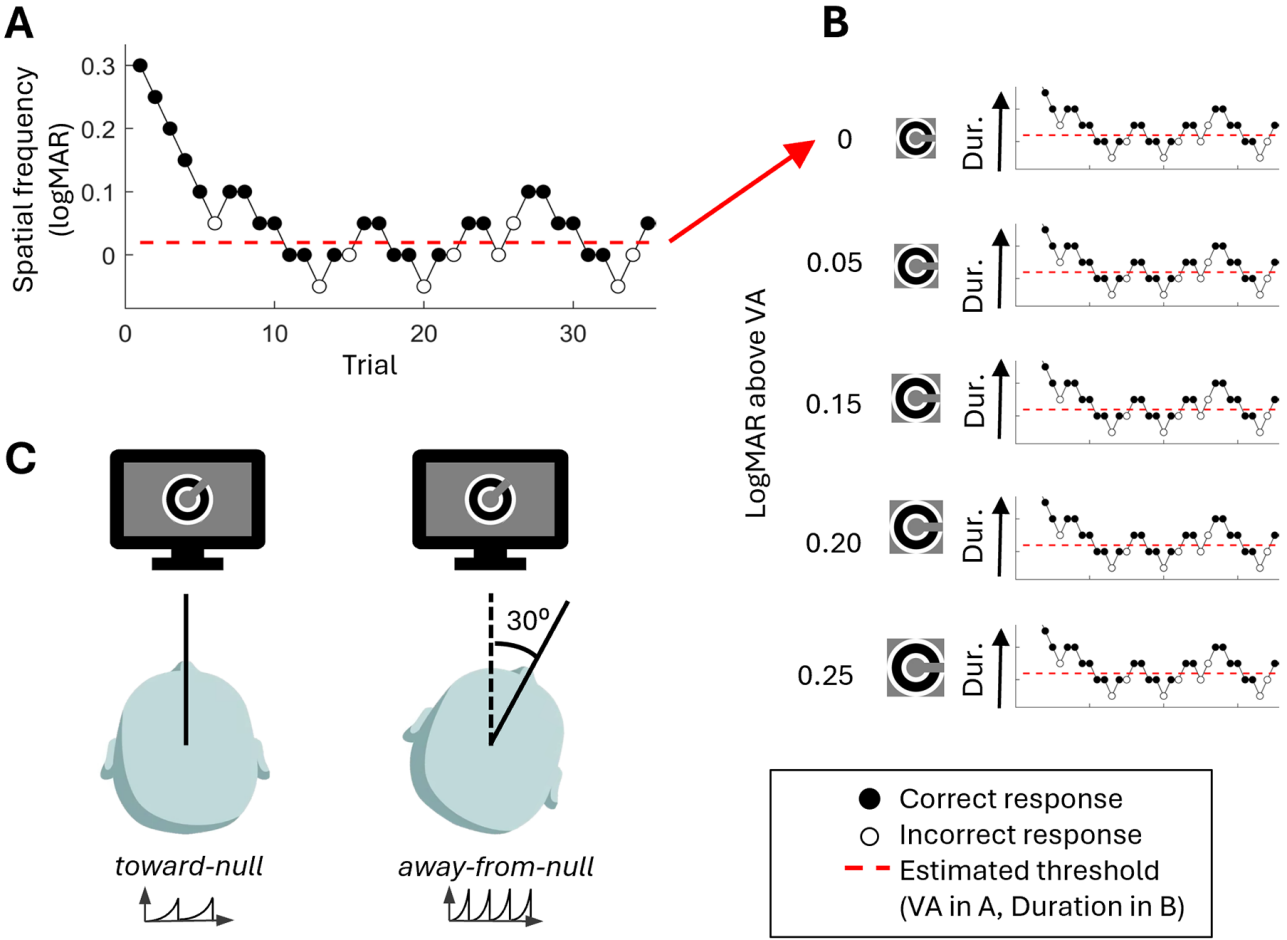
A schematic of the procedure for estimating duration thresholds. (**A**) A staircase procedure was used to estimate VA. (**B**) For different spatial frequencies relative to the estimated VA, staircases were used to estimate duration thresholds. (**C**) This procedure was repeated for both toward-null and away-from-null gaze positions. In this example, the away-from-null gaze position rotated the chin rest and head 30° to the right, resulting in the eyes rotating 30° to the left. See Methods for details on the staircase procedures.

A schematic of the protocol can be seen in Figure 3. The protocol had two phases. Phase one was estimation of VA, with spatial frequency being varied trial-by-trial according to the staircase procedure described above. Available spatial frequencies ranged from −0.30 to 1.75 logMAR, in 0.05-logMAR steps, with a small step size of 0.05 logMAR and a large step size of 0.15 logMAR. Each Landolt C stimulus was presented for 1000 ms. This duration was chosen to be above the critical duration required for VA in typically sighted individuals, as discussed in the introduction, as well as to encapsulate approximately three cycles of IN (given an average IN frequency of around 3 Hz^12^^,^^41^). Three repeats were performed, and the mean threshold was taken as VA. This phase was performed for both gaze positions in randomized order.

In the second phase, exposure duration threshold was estimated at five participant-relative spatial frequencies: VA from the worse performing gaze position in phase one and four 0.05-logMAR increments above. One staircase was run for each spatial frequency, in randomized order. Stimulus duration was varied according to the staircase procedure described above. The available stimulus durations were 40 points logarithmically spaced between 25 and 3000 ms, rounded to the nearest 25 ms (the smallest integer multiple of the 8.3-ms frame rate and the shortest increment displayed with reliable timing as confirmed using a camera recording at 120 Hz), with duplicate values removed. The small step size traversed the available values by two increments and the large step by five increments.

For both phases, all staircases for a single gaze position were performed together, then the gaze position was changed and the staircases for the other position were performed. The order of gaze position was randomized for each phase. Eccentric gaze positions were achieved for phases one and two by rotating the chair and chinrest and asking the patient to turn their eyes to view the monitor, as opposed to the patient's body facing straight ahead with only their eyes rotating. Eye movements recordings were attempted for all participants throughout phases one and two, although these are not discussed in the present paper.

### Calculating Psychophysical Thresholds

Thresholds for each staircase were calculated by averaging the last eight reversals. In a small subset of the exposure duration threshold staircases (typically occurring at the lowest spatial frequencies) (Table 2), truncation occurred before the full 12 reversals. This was because of a floor effect whereby the staircases quickly converged on the shortest available stimulus duration, leading to the alternative end criterion of 100 trials being reached. If this resulted in fewer than eight reversals, the threshold was calculated from all available reversals. This likely resulted in underestimation of threshold for these few staircases.

**Table 2.** Details of All Exposure Duration Staircases With Fewer Than 12 Reversals

	Spatial Frequency	
Participant Group	Toward Null	Away From Null
Control	+0.20	+0.20
Control	+0.15	N/A
Control	+0.15, +0.20	+0.20
IN	+0.20	N/A
IN	+0.05, +0.10, +0.15, +0.20	+0.15, +0.20

Each row represents one participant, with each entry representing the spatial frequency and gaze position at which the staircase did not reach the full 12 reversals. Spatial frequencies are relative to VA (logMAR).

### Statistical Analysis

Statistical analyses used linear mixed-effects models (LMMs) in JASP 0.95.4.^42^ All models included exposure duration threshold as the dependent variable and participant as a random effect. Independent variables included spatial frequency (modeled as nominal due to nonlinearities), gaze position, participant group, or nystagmus intensity (modeled as continuous). Models are described in the Results, and LMM ANOVA tables are reported in the Supplementary Materials (Supplementary Tables S1–S3). Post hoc pairwise comparisons were performed using contrasts of the estimated marginal means obtained from the LMMs, and *P* values were Holm–Bonferroni corrected.

## Results

**Figure 4.**
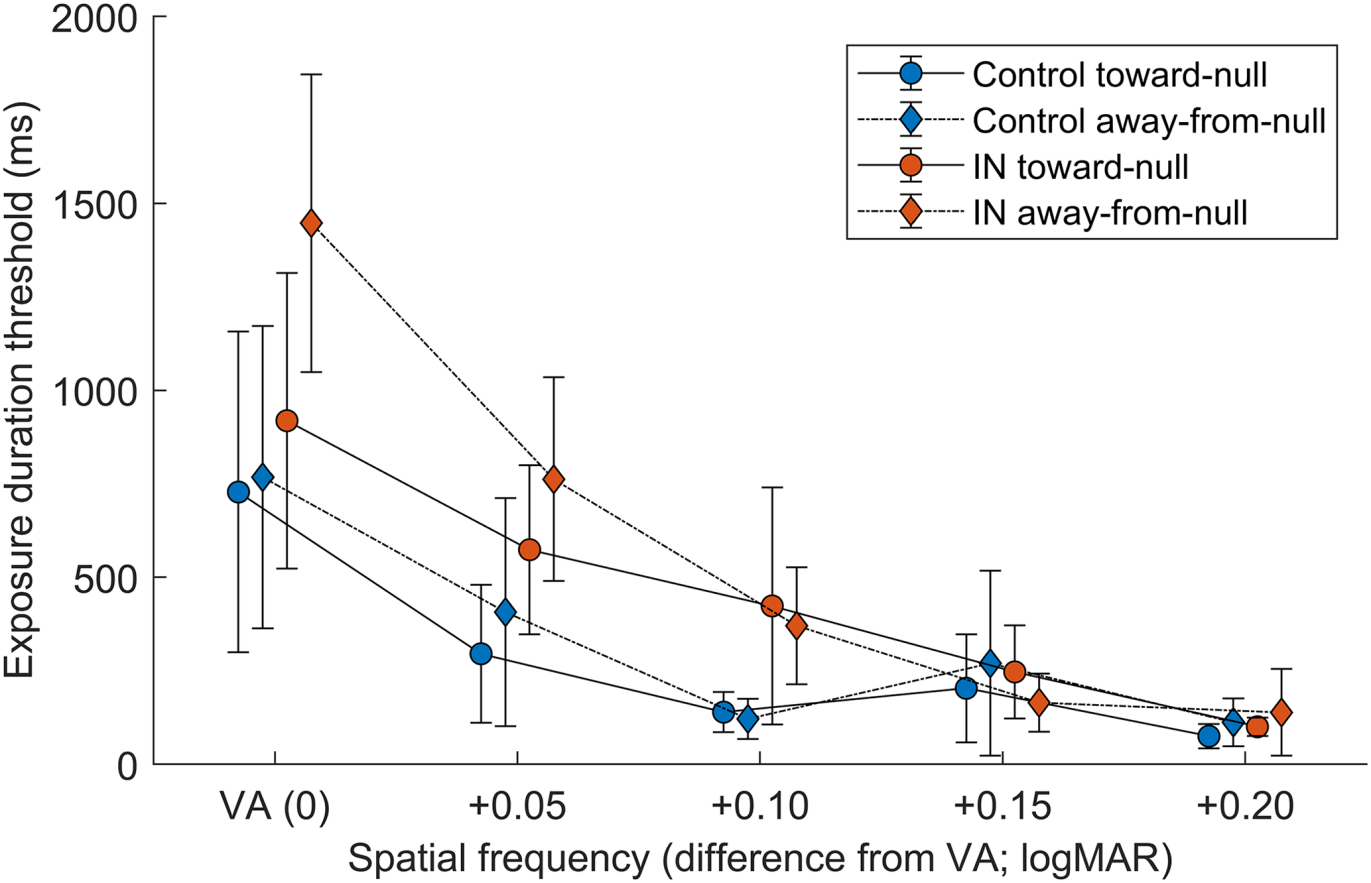
Mean stimulus exposure duration threshold as a function of spatial frequency, for both participant groups and both gaze positions tested. *Error bars* represent 95% confidence intervals. VA was determined for each participant in the first phase of the study, so it is effectively normalized across participants. Note that higher logMAR values indicate lower spatial frequencies.

Exposure duration thresholds for both participant groups and both gaze positions are plotted as a function of spatial frequency in Figure 4.

### Increased Exposure Duration Thresholds in IN

To test whether exposure duration thresholds were increased in IN compared to controls, a model with the following equation was used:

$$\begin{array}{ccc} & & \mathrm{Exposure}\;\mathrm{duration}\;\mathrm{threshold}∼\mathrm{spatial}\;\mathrm{frequency}\\ & & \;\times \mathrm{group}+\left(1|\mathrm{participant}\right) \end{array}$$

The ANOVA table for this model is provided as Supplementary Table S1. A significant main effect of group on exposure duration threshold was found (*F*_1,__30.00_ = 5.397, *P* = 0.027), as well as a significant interaction between group and spatial frequency (*F*_4,__280.00_ = 3.561, *P* = 0.007). Thus, the IN group required longer exposure durations than the control group overall. The difference between groups was larger at higher spatial frequencies (i.e., lower logMAR) (Fig. 4), and was statistically significant only at the level of VA (Supplementary Table S1). A significant main effect of spatial frequency was also observed, discussed further below.

### Exposure Duration Threshold Increases With IN Intensity

The hypothesis that exposure duration threshold is associated with nystagmus severity (as quantified by intensity) was tested using a model with the following equation:

$$\begin{array}{ccc} & & \mathrm{Exposure}\;\mathrm{duration}\;\mathrm{threshold}∼\mathrm{spatial}\;\mathrm{frequency}\\ & & \;\times \;\mathrm{intensity}+\left(1+\mathrm{intensity}|\mathrm{participant}\right) \end{array}$$

The ANOVA table for this model is provided as Supplementary Table S2. A significant main effect of IN intensity on exposure duration threshold was observed (*F*_1,__47.46_ = 4.769, *P* = 0.034), and the positive coefficient (mean ± SE β = 0.008 ± 0.004) of this effect reflects that exposure duration threshold increased with nystagmus intensity. A significant main effect of spatial frequency was again observed.

Because changes in intensity were elicited through changing gaze position, an additional model was used to validate that there was no significant main effect of gaze position in the control group. This served to confirm that the effect could be attributed to changes in intensity rather than other potential confounders from the use of eccentric head and eye positions, such as comfort. The equation for this model was as follows:

$$\begin{array}{ccc} & & \mathrm{Exposure}\;\mathrm{duration}\;\mathrm{threshold}∼\mathrm{spatial}\;\mathrm{frequency}\\ & & \;\times \mathrm{gaze}\;\mathrm{position}+\left(1|\mathrm{participant}\right) \end{array}$$

The ANOVA table for this model is provided as Supplementary Table S3. Although there was a significant effect of spatial frequency, no significant main effect of gaze position (*F*_1,__117.00_ = 0.578, *P* = 0.449) or interaction between spatial frequency and gaze position (*F*_4,__117.00_ = 0.114, *P* = 0.978) was found. Taken together, the above results demonstrate that exposure duration threshold depends on nystagmus exacerbation as quantified by intensity.

### Increased Exposure Duration Threshold With Spatial Frequency

Across all of the LMMs above, a significant main effect of spatial frequency was observed: *F*_4,280.00_ = 42.449, *P* < 0.001 (Supplementary Table S1); *F*_4,137.80_ = 7.837, *P* < 0.001 (Supplementary Table S2); *F*_4,__117.00_ = 14.240, *P* < 0.001 (Supplementary Table S3). The general trend across groups and gaze positions was that exposure duration threshold decreased as spatial frequency decreased (i.e., less time was needed to correctly resolve larger, less detailed stimuli). As discussed above, a significant interaction was observed between spatial frequency and group (*F*_4,280.00_ = 3.561, *P* = 0.007) (Supplementary Table S1). This highlights that, although the overall trend is similar between the two groups, the effect does differ slightly, with the control group asymptoting at +0.10 logMAR above VA whereas a downward trend was present in the IN group across all spatial frequencies tested.

## Discussion

This study examined how exposure duration threshold for resolution of a vanishing optotype is influenced by spatial frequency as well as the presence and severity of IN. Duration thresholds were found to be significantly higher in individuals with IN compared to typically sighted controls, and, for those with IN, they were higher under conditions of greater IN intensity. These results demonstrate increased “time to see” in IN, suggesting that exposure duration threshold could be a useful measure of visual function in people with IN.

### Increased Exposure Duration Thresholds in IN

The finding that exposure duration thresholds are higher in IN than controls agrees with previous reports of increased response times in IN^15^^,^^16^ and confirms that the effect exists at a perceptual level, independent of motor response. Notably, the differences between groups were greater for stimuli with higher spatial frequencies and minimal for stimuli over 0.10 logMAR above maximum VA. Therefore, “time to see” becomes especially important for stimuli approaching an individual's VA limit.

The fact that “time to see” has greater impact for stimuli approaching VA is not unexpected when one considers the interplay between the spatial properties of the stimulus and fixation in IN, given that delayed recognition time in IN is known to be at least partly due to delays in acquiring fixation.^18^ For optotype stimuli, size and spatial frequency are coupled; that is, stimuli with lower spatial frequencies are physically larger. Larger stimuli may require less precise fixation than VA-level stimuli, potentially reducing any delay in fixation. Additionally, less fixation time may be required to resolve lower spatial frequencies, reducing the need for multiple foveation periods and hence the overall required exposure time. At lower spatial frequencies, mean duration thresholds were around 270 ms or less and comparable across groups. The mean nystagmus cycle frequency among our IN sample was 3.6 Hz (corresponding to a cycle duration of 276 ms), suggesting that stimuli resolvable within a single IN cycle do not require additional exposure time in IN relative to controls.

### Exposure Duration Threshold Increases With IN Intensity

The finding that exposure duration thresholds were higher under conditions of exacerbated IN (i.e., increased IN intensities) aligns with previous findings of longer response times when IN intensity is increased due to stress,^16^ as well as time-limited VA being reduced at eccentric gaze positions^27^ (at which IN intensity is presumably increased, given that the null zone tends to lie within 10° of the primary position). This result has important implications for exposure duration as an outcome measure. A key requirement for outcome measures in IN is sensitivity to within-individual waveform changes. In the present study, mean VA in the IN group differed by less than 0.02 logMAR between gaze positions (0.692 toward-null vs. 0.706 away-from-null, an increase of only 2%), whereas mean duration threshold at VA differed by 528 ms (919 toward-null vs. 1447 away-from-null, an increase of 57%). This suggests that exposure duration may be notably more sensitive to changes in IN intensity than the conventional measure of VA. Given that the effect of IN intensity on exposure duration threshold was largest at participant-relative VA and 0.05 logMAR above (Supplementary Table S2), future work in this area should focus specifically on stimuli at the level of VA to further investigate the suitability of exposure duration threshold as an outcome measure in IN.

### Increased Exposure Duration Threshold With Spatial Frequency

The increase in exposure duration threshold with spatial frequency observed in control participants is consistent with previous findings of better VA being achieved with longer presentation durations.^3^^–^^11^ This agreement was expected because the same relationship (between spatial frequency and stimulus duration) is examined, although this study measured the exposure duration required to resolve a given spatial frequency whereas previous works measured the spatial frequency resolvable for a given duration. Although it is difficult to make direct comparisons with these prior studies due to methodological and stimulus differences, if one assumes critical duration to occur at VA, beyond which longer presentation is of little benefit, the exposure duration threshold found in this study for control participants at VA (∼700 ms) aligns with the 251- to 1000-ms range reported previously.

The relationship between spatial frequency and presentation duration has been less well characterized in IN, with this study being the first, to our knowledge, to examine how duration threshold varies over a series of spatial frequencies. The similarity in trend between those with IN and controls is a novel albeit anticipated, finding. Comparison can be made with Elkamshoushy et al.,^29^ who reported shorter recognition times for optotypes presented 0.10 logMAR above VA compared to at VA in individuals with idiopathic IN, indicating that exposure duration threshold decreases with decreased spatial frequency, as demonstrated by the present findings.

### Factors Contributing to “Time to See”

Two main factors likely contribute to increased “time to see” in IN. First, associated visual impairments, such as comorbid pathology or amblyopia, may increase critical duration (as seen in conditions such as retinitis pigmentosa,^6^ central serous retinopathy, macular edema, and glaucoma^4^). Similar effects may occur in other conditions commonly associated with IN. The increased exposure duration thresholds observed for stimuli with higher spatial frequencies in this study may also apply to stimuli that are challenging to resolve for other reasons, such as reduced VA due to refractive blur or pathology.

The second factor is the IN oscillation itself. The present study focused on IN intensity, which is a widely used metric of IN severity. Numerous other waveform characteristics, including amplitude, frequency, and foveation characteristics (which can be represented by algorithmic metrics such as the expanded nystagmus acuity function [NAFX]^22^), also have a known relationship with factors affecting IN severity.^16^^,^^21^^,^^43^^,^^44^ Because highest resolution is typically achieved at the fovea, accurate discrimination of a stimulus might be reached most quickly with a waveform that maximizes total foveation time (i.e., long duration and high positional stability of the foveation period), which would minimize the number of cycles required to accrue sufficient visual information. This would be consistent with the finding of Jones et al.^16^ that reduced foveation duration is associated with increased response time when IN intensity is increased through stress. However, the relationship between the foveation period and other aspects of the waveform is complex. For example, Jones et al.^16^ also reported an association between shorter foveation periods and increases in amplitude and intensity in response to stress, which in turn might influence exposure threshold duration. Thus, the relationship between IN intensity and exposure duration threshold found in the present study could alternatively be expressed in terms of other waveform characteristics.

The size and spatial frequency of a stimulus affect resolvability by a moving eye, regardless of waveform characteristics. Stimulus size and spatial frequency are coupled in the optotypes used for this study, but their individual effects should also be considered. The image of a smaller stimulus may be displaced from the fovea by the IN oscillation, whereas a larger stimulus may remain partially within the foveal region, allowing more information to be gathered within a single cycle. The nature of perception throughout the waveform is also relevant: If vision is suppressed outside the foveation period, size effects may only apply when a stimulus is smaller than foveation amplitude. If not, retinal image blur during the accelerating slow phase may become more relevant, particularly for higher spatial frequencies.

One can also consider the effect of the resolution profile of the fovea and surrounding areas. Abnormal foveal development, such as foveal hypoplasia, is common in IN and associated conditions.^45^ The fovea has also been shown to be relatively horizontally elongated in people with IN,^46^ although it is not yet known whether this elongation is due to redistribution of the typical number of photoreceptors over a larger area, which might suggest an adaptation to maximize resolution over time. Such flattening and broadening of the retinal resolution profile could potentially increase the area over which stimuli can be resolved. This might reduce the need for precise fixation and allow useful visual information to be gathered throughout a greater proportion of the waveform, reducing the effect of the oscillation on exposure duration threshold.

The present study investigated a specific aspect of visual function, whereas real-world vision involves larger and far more complex stimuli that may require multiple fixations. Other factors also influence task difficulty. Real-world stimuli are often in motion, and velocity discrimination and coherent motion perception are known to be impaired in IN.^35^^,^^36^ Contrast sensitivity, which improves with longer stimulus duration in IN much like VA,^47^ will also be impactful. Future work examining whether waveform changes affect the time required for real-world activities such as reading and face recognition, which are known to be slower in IN than in controls,^48^^,^^49^ will contribute to an improved understanding of overall visual function in IN.

### Implications of “Time to See”

Our findings have clear implications concerning the everyday visual experience of those with IN: Maximum VA can be achieved when there is no restriction to viewing time, but the introduction of time limits will have a deleterious effect on resolving ability. This is further relevant to routine clinical measurement of VA, as those with IN may require more viewing time to read a letter chart at the level of VA. Although the optotype in this experiment differs from the standard optotypes typically used in clinical practice, a minimum of 2 seconds of viewing time per letter may nonetheless be an appropriate recommendation. A further implication of the present findings relates to the visual benefits of the null zone, with the greater “time to see” found when viewing away from the null zone under conditions of increased nystagmus intensity likely being a contributing factor to the preferential use of the null zone by those with IN.

## Conclusions

In summary, exposure duration thresholds were increased in participants with IN compared to controls at participant-relative VA and were seen to increase under conditions of exacerbated IN (increased intensity). These findings demonstrate the potential of “time to see” as a measure of vision that reflects changes in visual function resulting from modification of the IN oscillation. An appreciation of the temporal aspect of perception is likely to aid in the development of improved clinical measures for assessment of visual function and evaluation of visual outcomes of IN-dampening treatments and interventions.

## Supplementary Material

Supplement 1
