# Supplementary material for: “Time to See” in Infantile Nystagmus
Source: Invest Ophthalmol Vis Sci. 2026 May 7;67(5):17. doi: 10.1167/iovs.67.5.17 (PMC13170726; doi:10.1167/iovs.67.5.17)
Supplement: Supplement 1 [file iovs-67-5-17_s001.docx]

# Supplementary Material

Supplementary Table S1. Results of the LMM examining the effects of participant group and spatial frequency on exposure duration threshold.

| *ANOVA Summary* | | | | | | |  |
| --- | --- | --- | --- | --- | --- | --- | --- |
| Effect | | | df | F | | p |  |
| Spatial frequency | | | 4, 280.00 | 42.449 | | < .001 |  |
| Group | | | 1, 30.00 | 5.397 | | .027 |  |
| Spatial frequency ×  Group | | | 4, 280.00 | 3.561 | | .007 |  |
|  | | | | | | |  |
|  | | | | | | |  |
| Note: Type III Sum of Spheres | | | | | | |  |
| *Post hoc pairwise comparisons – Spatial frequency* *× Group - contrasting control (1) with IN (-1)* | | | | | | | |
|  | | | | 95% CI | |  | |
| Spatial frequency | Estimate | SE | df | Lower | Upper | z | P_holm_ |
| VA | -0.435 | 0.127 | ∞ | -0.686 | -0.184 | -3.393 | .003 |
| VA + 0.05 logMAR | -0.317 | 0.127 | ∞ | -0.568 | -0.066 | -2.475 | .053 |
| VA + 0.10 logMAR | -0.267 | 0.127 | ∞ | -0.518 | -0.015 | -2.080 | .113 |
| VA + 0.15 logMAR | 0.031 | 0.127 | ∞ | -0.220 | 0.282 | 0.242 | 1.000 |
| VA + 0.20 logMAR | -0.026 | 0.127 | ∞ | -0.277 | 0.225 | -0.202 | 1.000 |
|  | | | | | | | |
| Note: p-values are adjusted using Holm correction. | | | | | | | |

Supplementary Table S2. Results of the LMM examining the effects of IN intensity and spatial frequency on exposure duration threshold in participants with IN.

| *ANOVA Summary* | | | | |
| --- | --- | --- | --- | --- |
| Effect | df | F | | p |
| Spatial frequency | 4, 137.80 | 7.837 | | <.001 |
| Intensity | 1, 47.46 | 4.769 | | .034 |
| Spatial frequency  *×*  Intensity | 4, 137.80 | 1.633 | | .169 |
|  | | | | |
| Note: Type III Sum of Spheres  *Estimated Trends* *(Spatial frequency × Intensity)* | | | | |
|  | | | 95% CI | |
| Spatial frequency | Intensity (slope) | SE | Lower | Upper |
| VA | 0.016 | 0.006 | 0.003 | 0.029 |
| VA + 0.05 logMAR | 0.017 | 0.006 | 0.004 | 0.029 |
| VA + 0.10 logMAR | 4.244×10^-4^ | 0.006 | -0.012 | 0.013 |
| VA + 0.15 logMAR | 0.003 | 0.006 | -0.010 | 0.016 |
| VA + 0.20 logMAR | 0.004 | 0.006 | -0.009 | 0.016 |
|  | | | | |

Note: Type III Sum of Spheres

Supplementary Table S3. Results of the LMM examining the effects of gaze position and spatial frequency on exposure duration threshold in control participants.

| *ANOVA Summary* | | | |
| --- | --- | --- | --- |
| Effect | df | F | p |
| Spatial frequency | 4, 117.00 | 14.240 | < .001 |
| Gaze position | 1, 117.00 | 0.578 | .449 |
| Spatial frequency  *  Gaze position | 4, 117.00 | 0.114 | .978 |
|  | | | |

Note: Type III Sum of Spheres
